# Efficacy and Safety of Adding Immune Checkpoint Inhibitors to Neoadjuvant Chemotherapy Against Triple-Negative Breast Cancer: A Meta-Analysis of Randomized Controlled Trials

**DOI:** 10.3389/fonc.2021.657634

**Published:** 2021-11-29

**Authors:** Yunhai Li, Lei Xing, Fan Li, Hong Liu, Lu Gan, Dejuan Yang, Mengxue Wang, Xuedong Yin, Hongyuan Li, Guosheng Ren

**Affiliations:** ^1^ Department of Endocrine and Breast Surgery, The First Affiliated Hospital of Chongqing Medical University, Chongqing, China; ^2^ Department of Oncology, The First Affiliated Hospital of Chongqing Medical University, Chongqing, China; ^3^ Chongqing Key Laboratory of Molecular Oncology and Epigenetics, The First Affiliated Hospital of Chongqing Medical University, Chongqing, China

**Keywords:** triple-negative breast cancer (TNBC), neoadjuvant chemotherapy, immune checkpoint inhibitors (ICI), pathological complete response, meta-analysis

## Abstract

**Background:**

Immune checkpoint inhibitors (ICIs) have shown promising anti-tumor activity in multiple malignances including breast cancer. However, the responses can vary. This meta-analysis was conducted to evaluate the efficacy and safety profile of adding ICIs to neoadjuvant chemotherapy against triple-negative breast cancer (TNBC) and assess correlation of PD-L1 tumor status with responses.

**Methods:**

Eligible studies were retrieved from the PubMed, Embase, and Web of Science databases. Randomized controlled trials (RCTs) that investigated ICI-containing *versus* ICI-free neoadjuvant therapy were included in this study. Meta-analyses were performed using Review Manager Version 5.2 software.

**Results:**

This study included four RCTs containing 1795 patients with early TNBC. Compared with ICI-free neoadjuvant therapy, ICI-containing neoadjuvant therapy significantly increased the pathological complete response (pCR) rates in TNBC (odds ratio [OR] = 2.14, 95% confidence interval [CI]: 1.37–3.35, *P* < 0.001). In subgroup analysis, the addition of ICI to neoadjuvant chemotherapy was significantly associated with increased pCR rate in both PD-L1-positive TNBC (OR = 1.79, 95% CI: 1.33–2.41, *P* < 0.001) and PD-L1-negative TNBC (OR = 1.84, 95% CI: 1.14–2.99, *P* = 0.01). Patients with TNBC receiving ICI-containing neoadjuvant therapy had a better event-free survival (hazard ratio = 0.66, 95% CI: 0.48–0.89, *P* = 0.007) than those who receiving ICI-free neoadjuvant therapy. A significantly higher risk of adverse events including adrenal insufficiency, increased aspartate aminotransferase, dry skin, hepatitis, hyperthyroidism, hypothyroidism, infusion related reaction, pyrexia, and stomatitis was associated with ICI-containing neoadjuvant therapy.

**Conclusion:**

ICI-containing neoadjuvant therapy significantly increased the pCR rate in TNBC patients, independently of PD-L1 status. The addition of ICI to neoadjuvant chemotherapy may be considered an option for TNBC patients.

## Introduction

Neoadjuvant treatment is widely used to reduce the size and extent of tumors in high risk early breast cancer (BC). Patients who achieve a pathological complete response (pCR) after neoadjuvant therapy have better survival outcomes than those with residual invasive disease (1). Current neoadjuvant treatment strategies include chemotherapy, anti-human epidermal growth receptor 2 (HER2) therapy, endocrine therapy, and co-administration for different BC subtypes. Due to the lack of anti-HER2 therapy and potential antagonism between endocrine therapy and chemotherapeutic agents, anthracycline plus cyclophosphamide- and taxane-based neoadjuvant chemotherapy remains the major choice for patients with triple-negative BC (TNBC) (2, 3). Following standard neoadjuvant chemotherapy, only approximately 30% of patients with TNBC achieve pCR (1). Considering a significant association between pCR and favorable survival outcomes (4), new strategies and agents are urgently needed to further increase the pCR rates in patients with TNBC.

Immune-checkpoint therapy targeting the programmed cell death protein 1 (PD-1)/programmed death-ligand 1 (PD-L1) axis is a promising strategy for several malignances (5). With the major advancements of agents targeting the PD-1/PD-L1 axis, multiple immune checkpoint inhibitors (ICIs) have been shown to be effective against different advanced solid tumors and hematological malignancies, such as melanoma, non-small cell lung cancer, and gastric carcinoma (6). In BC, IMpassion130 trial demonstrated that atezolizumab combined with nab-paclitaxel significantly improve progression-free survival (PFS) and overall survival (OS) in patients with metastatic TNBC and PD-L1-positive subgroup with acceptable safety profile (7). Although pembrolizumab showed promising anti-tumor activities and safety, there was no significant PFS benefit for patients with HER2-negative metastatic BC (8–10). However, new evidence has indicated that the addition of pembrolizumab to standard neoadjuvant chemotherapy markedly improves the pCR rate in early HER2-negative BC and TNBC (11, 12). These findings support further investigation into the addition of ICIs to neoadjuvant therapy in TNBC.

To provide up to date evidence on this emerging topic, we performed a meta-analysis of randomized controlled trials (RCTs) to assess the efficacy and safety of adding ICIs to neoadjuvant chemotherapy in early TNBC.

## Methods

### Search Strategy and Study Identification

Literatures published before October 01, 2020 were retrieved from the PubMed, Embase, and Web of Science databases with the use of the following keywords: immune checkpoint inhibitors, nivolumab, pembrolizumab, ipilimumab, avelumab, tremelimumab, atezolizumab, durvalumab, and TNBC without further restrictions. The citation lists of relevant studies, reviews, and meta-analyses were manually screened for potentially eligible publications. The literature search was independently performed by two of the authors (LYH and XL). Any discrepancy was solved by discussion with a third author (YXD).

### Selection Criteria

The inclusion and exclusion criteria were prespecified. Eligible studies had to satisfy the following criteria: (a) phase II or phase III RCTs; (b) RCTs including early TNBC patients who received ICI-containing neoadjuvant therapy in the experimental arm and ICI-free neoadjuvant therapy in the control arm; and (c) RCTs with available data on pCR rates in the experimental and control arms for the estimation of an odds ratio (OR) and 95% confidence interval (CI). Studies were excluded if they were: (a) non-RCTs conducted to evaluate the role of ICI-containing neoadjuvant therapy in TNBC patients; (b) single-arm studies; (c) studies to determine appropriate dosages; and (d) ongoing trials or abstracts with insufficient results. If multiple publications from the same trial were identified or if there was case overlap between publications, only the latest or most complete publication was included. Two reviewers (LYH and LF) independently evaluated the risk of bias of the eligible studies using the Cochrane Collaboration risk of bias tool (13).

### Data Extraction

Data were independently extracted by two of the authors (LYH and XL). The following data obtained from the eligible studies were recorded in accordance with a prespecified protocol: name of the trial, year of publication, study design, number of randomized patients, details of neoadjuvant therapy regimens administered, number of patients achieving pCR, follow-up information, and number of adverse events (AEs). Hazard ratio (HR) and 95% CI of event-free survival (EFS), OS, and distant recurrence-free survival were extracted when available. If not reported, the HRs and associated statistical data were indirectly calculated using the methods reported by Parmar (14) with an Excel spreadsheet (version 3.0, September 28, 2004) developed by Sydes and Tierney in collaboration with the MRC Clinical Trials Unit (London, England).

### Definition of Outcomes

The primary objective of this study was to compare the efficacy of ICI-containing neoadjuvant therapy *versus* ICI-free neoadjuvant therapy in TNBC patients, in terms of pCR, which was defined as the absence of invasive tumors in the breast and regional nodes at the time of surgery (ypT0/is pN0). If not reported, other definitions of pCR (ypT0 ypN0 and ypT0/is) were substituted. The secondary objectives were as follows (1): the EFS for patients who experienced disease progression, local or distant recurrence, developing a second primary tumor, or death; and (2) the number of patients who had AEs for all grades and grade 3 or higher. We hypothesized that there was no significant correlation between molecular subtypes and AEs. Therefore, if the AEs of patients with TNBC were not available, we included the number of AEs from all molecular subtypes of breast cancer patients.

### Statistical Analysis

ORs and 95% CIs were calculated for pCR and AEs. An OR > 1 indicated higher pCR and AEs rates, whereas an OR < 1 indicated lower pCR and AEs rates in the ICI-containing group than in the ICI-free group. The HR with 95% CI was calculated to estimate the impact of ICI-containing neoadjuvant therapy on survival outcomes. A HR > 1 indicated worse survival outcomes, whereas a HR < 1 indicates better survival outcomes in the ICI-containing group compared with the ICI-free group. Heterogeneity was assessed using the Cochran Q and *I* ^2^ statistics, and a *P* < 0.10 or *I* ^2^ statistic > 50% was considered to indicate substantial heterogeneity. Clinical heterogeneity (e.g., full characteristics of participants and treatment details) and methodological heterogeneity (e.g., randomization process, drugs, and blinding method) were regarded as potential source of heterogeneity. A random-effects model was used by default due to potential clinical or methodological heterogeneity, or both in the included studies. The Mantel-Haenszel method was used to calculate pooled ORs with corresponding 95% CIs. Pooled HRs and corresponding 95% CIs were calculated using the inverse variance method. All analyses were performed using Review Manager 5.2 software (The Nordic Cochrane Center, Cochrane Collaboration, Copenhagen, Denmark). The pooled ORs and HRs were considered statistically significant if the 95% CI did not include 1.0 with a *P <*0.05 (two-sided).

## Results

### Literature Search and Study Characteristics

**Figure 1:**
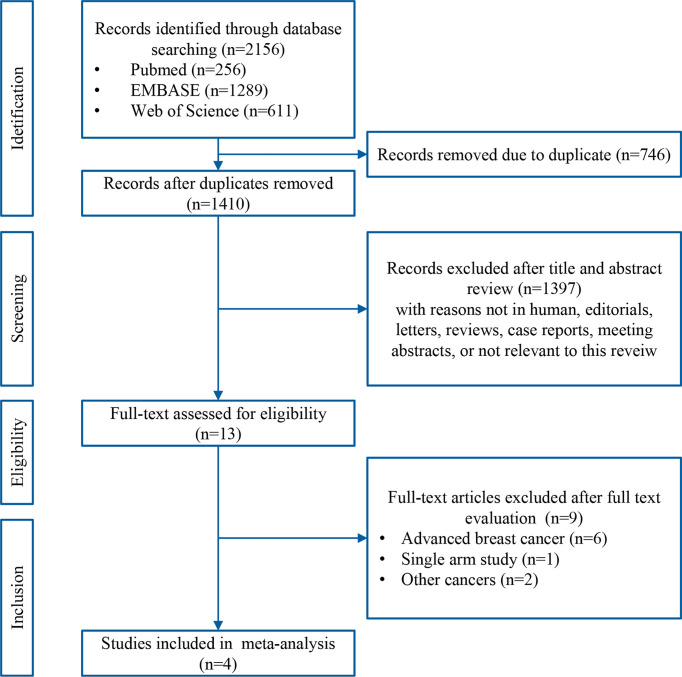
Flow chart of the literature search and study selection.

A systematic search of the literature identified 2156 records. After removing duplicates, the titles and abstracts of the remaining 1410 records were screened, and 1397 non-relevant records were excluded. Thirteen potentially eligible articles were evaluated in greater detail, of which nine did not met the eligibility criteria for this study. Finally, four RCTs [GeparNuevo (15), I-SPY2 (12), IMpassion031 (16), and KEYNOTE-522 (11)] were included in this meta-analysis. A flow chart of the literature search and selection process is presented in **Figure 1**.

A total of 1795 patients with TNBC were included in the study, of whom 1066 (59.4%) received ICI-containing and 729 (40.6%) received ICI-free neoadjuvant therapy. The four RCTs were published between 2019 and 2020. All patients were enrolled between 2015 and 2018 from multicenter. There were two phase II studies (GeparNuevo and I-SPY2) and two phase III studies (IMpassion031 and KEYNOTE-522). The GeparNuevo, IMpassion031, and KEYNOTE-522 trials included only TNBC patients, whereas the I-SPY2 trial included both TNBC and hormone receptor-positive/HER2-negative BC patients. Taxane and/or anthracycline plus cyclophosphamide were included in the neoadjuvant regimens in the four RCTs, whereas the agents in the KEYNOTE-522 trial also contained carboplatin. Durvalumab and atezolizumab were added to the neoadjuvant chemotherapy in the GeparNuevo and IMpassion031 trials, respectively. Pembrolizumab was added to the neoadjuvant chemotherapy in the KEYNOTE-522 and I-SPY2 trials. A placebo was given to the control group in the GeparNuevo, IMpassion031, and KEYNOTE-522 trials. The main characteristics of the four RCTs are presented in **Table 1**. The results of quality assessment are shown in **Supplementary Figure S1**.

**Table 1:** Main characteristics of the included randomized controlled trials.

Study	Year	Trial design	Treatment arms	Primary end points	Secondary end points	No. of TNBC patients
GeparNuevo^15^	2019	Multicenter, phase II	Durvalumab+ CT^a^	pCR^e^	pCR^f, g^; PD-L1^h^	88
			Placebo+CT^a^			86
I-SPY2^12^	2020	Multicenter, phase II	Pembrolizumab+CT^b^	pCR^f^	RCB; EFS; DRFS	29
			CT^b^			85
IMpassion031^16^	2020	Multicenter, phase III	Atezolizumab+CT^c^	pCR^f^	EFS; OS PD-L1^h^	165
			Placebo+CT^c^			168
KEYNOTE-522^11^	2020	Multicenter, phase III	Pembrolizumab+CT^d^	pCR^f^; EFS	pCR^g^; PD-L1^h^; OS	784
			Placebo+CT^d^			390

CT, chemotherapy; pCR, pathologic complete response; EFS, event-free survival; PD-L1, programmed cell death-ligand 1; OS, overall survival; RCB, residual cancer burden; DRFS, distant recurrence-free survival; TNBC, triple-negative breast cancer.

a Durvalumab (750mg) or placebo monotherapy 2 weeks before start of chemotherapy followed by durvalumab (1500mg) or placebo once every 4 weeks plus nab-paclitaxel 125 mg/m^2^ weekly for 12 weeks, followed by durvalumab (1500mg) or placebo once every 4 weeks plus epirubicin/cyclophosphamide once every 2 weeks for 4 cycles.

b Pembrolizumab (200 mg) concurrently with paclitaxel in weeks 1, 4, 7, and 10 (4 cycles). Paclitaxel 80 mg/m^2^ weekly for 12 weeks, followed by doxorubicin 60 mg/m^2^ plus cyclophosphamide 600 mg/m^2^ once every 2 to 3 weeks for 4 cycles. No placebo was given in the control group.

c Atezolizumab (840 mg) or placebo once every 2 weeks combined with nab-paclitaxel 125 mg/m² once per week for 12 weeks, followed by atezolizumab (840 mg) or placebo combined with doxorubicin 60 mg/m² and cyclophosphamide 600 mg/m² once every 2 weeks for 4 cycles.

d Pembrolizumab (200mg) or placebo once every 3 weeks plus paclitaxel 80 mg/m^2^ once weekly plus carboplatin area under curve 5 once every 3 weeks or 1.5 once weekly in the first 12 weeks, followed by pembrolizumab (200mg) or placebo once every 3 weeks plus doxorubicin 60 mg/m^2^ or epirubicin 90 mg/m^2^ plus cyclophosphamide 600 mg/m^2^ once every 3 weeks in the subsequent 12 weeks.

e The pCR was defined as the absence of residual invasive and in situ in breast and regional nodes (ypT0 ypN0).

f The pCR was defined as the absence of invasive tumor in breast and regional nodes (ypT0/Tis ypN0).

g The pCR including ypT0 ypN0, the absence of invasive tumor in breast (ypT0/Tis), the absence of residual invasive and in situ in breast (ypT0), and the absence of residual invasive and in situ in regional nodes (ypN0).

h The defined pCR for patients with PD-L1 status information.

### The pCR Rates

**Figure 2:**
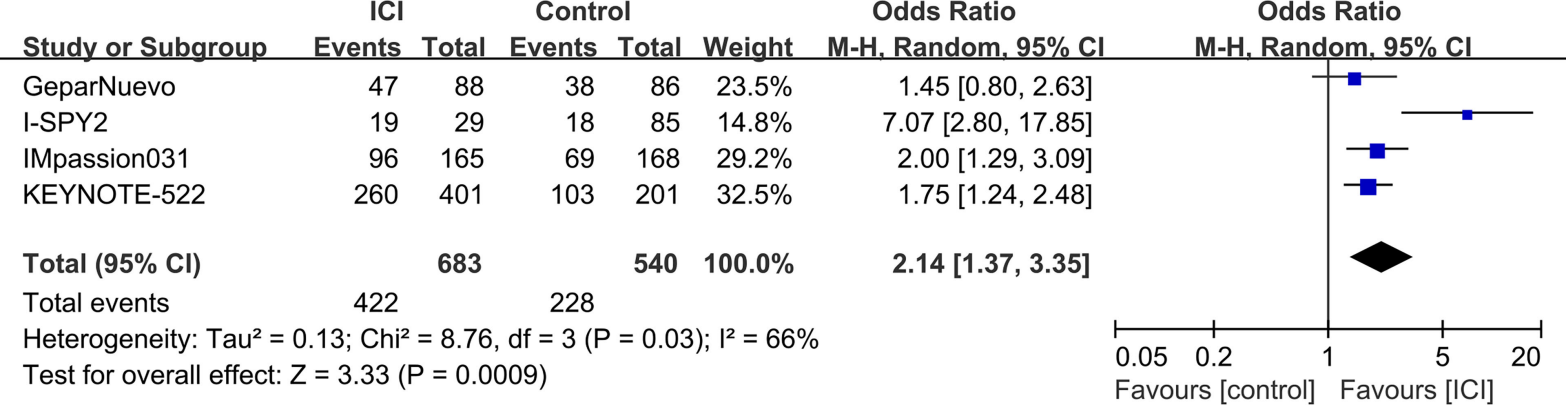
Forest plots of meta-analyses of pathological complete response (pCR). Immune checkpoint inhibitor (ICI)-containing neoadjuvant therapy compared with ICI-free neoadjuvant therapy for triple-negative breast cancer (TNBC).

**Figure 3:**
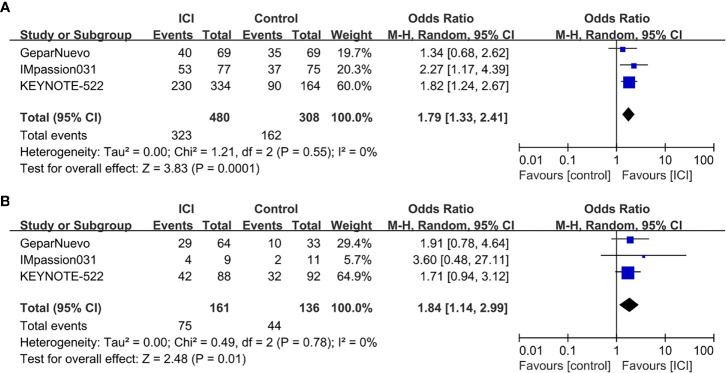
Forest plots of subgroup meta-analyses of pCR based on PD-L1 status. **(A)** ICI-containing neoadjuvant therapy compared with ICI-free neoadjuvant therapy in TNBC patients with PD-L1-positive tumors. **(B)** ICI-containing neoadjuvant therapy compared with ICI-free neoadjuvant therapy in TNBC patients with PD-L1-negative tumors.

The pCR rates were analyzed for 1223 TNBC patients. Overall, 422 (61.8%) of 683 patients in the ICI-containing group and 228 (42.2%) of 540 patients in the ICI-free group achieved a pCR after neoadjuvant treatment (OR = 2.14, 95% CI: 1.37–3.35, *P* < 0.001; heterogeneity: *I* ^2^ = 66%, *P* = 0.03; **Figure 2**). Subgroup analyses were performed according to anti-PD-1 (KEYNOTE-522 and I-SPY2) and anti-PD-L1 (GeparNuevo and IMpassion031) inhibitors; 279 (64.9%) of 430 patients in the anti-PD-1-containing group and 121 (42.3%) of 286 patients in the control group achieved a pCR (OR = 3.29, 95% CI: 0.84–12.80, *P* = 0.09; heterogeneity: *I* ^2^ = 87%, *P* = 0.006; **Supplementary Figure S2A**), whereas 143 (56.5%) of 253 patients in the anti-PD-L1-containing group and 107 (42.1%) of 254 patients in the control group achieved a pCR (OR = 1.79, 95% CI: 1.26–2.54, *P* = 0.001; heterogeneity: *I* ^2^ = 0%, *P* = 0.39; **Supplementary Figure S2B**). Three of the four studies (GeparNuevo, IMpassion031, and KEYNOTE-522) reported pCR data based on PD-L1 status. Subgroup analyses were performed by stratifying patients into PD-L1-positive and -negative groups. Among TNBC patients with PD-L1-positive tumors, 323 (67.3%) of 480 patients in the ICI-containing group and 162 (52.6%) of 308 patients in the ICI-free group achieved a pCR (OR = 1.79, 95% CI: 1.33–2.41, *P* < 0.001; heterogeneity: *I* ^2^ = 0%, *P* = 0.55; **Figure 3A**). In the PD-L1-negative subgroup, 75 (46.6%) of 161 patients in the ICI-containing group and 44 (32.4%) of 136 patients in the ICI-free group achieved a pCR (OR = 1.84, 95% CI: 1.14–2.99, *P* = 0.01; heterogeneity: *I* ^2^ = 0%, *P* = 0.78; **Figure 3B**). The PD-L1-positive subgroup achieved a higher pCR rate than the PD-L1-negative subgroup in TNBC patients receiving not only ICI-containing neoadjuvant therapy (OR = 2.48, 95% CI: 1.67–3.69, *P* = 0.01; heterogeneity: *I* ^2^ = 0%, *P* = 0.85; **Supplementary Figure S3A**), as well as ICI-free neoadjuvant therapy (OR = 2.29, 95% CI: 1.43–3.67, *P* < 0.001; heterogeneity: *I* ^2^ = 0%, *P* = 0.47; **Supplementary Figure S3B**).

### EFS

**Figure 4:**
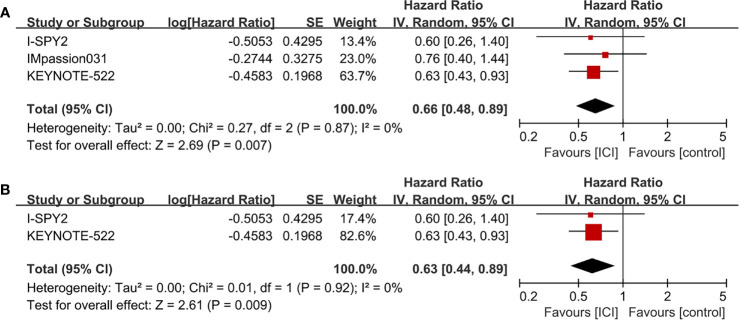
Forest plots of meta-analyses for event-free survival (EFS). **(A)** ICI-containing neoadjuvant therapy compared with ICI-free neoadjuvant therapy for TNBC. **(B)** Anti-PD-1-containing neoadjuvant therapy compared with ICI-free neoadjuvant therapy for TNBC.

The median follow-up periods were ranged from 15.5 to 42.0 months in the three RCTs (I-SPY, IMpassion031, and KEYNOTE-522) with EFS information. The pooled data showed that ICI-containing neoadjuvant therapy was significantly associated with a better EFS (HR = 0.66, 95% CI: 0.48–0.89, *P* = 0.007; heterogeneity: *I* ^2^ = 0%, *P* = 0.87) than ICI-free neoadjuvant therapy in TNBC patients (**Figure 4A**). In subgroup analysis, patients receiving anti-PD-1-containing neoadjuvant therapy had a better EFS (HR = 0.63, 95% CI: 0.44–0.89, *P* = 0.009; heterogeneity: *I* ^2^ = 0%, *P* = 0.92) than the control group (**Figure 4B**).

### AEs

There were 64 types of all-grade AEs reported by at least two of the four RCTs and were available for meta-analysis. The pooled effects for all-grade AEs showed that ICI-containing neoadjuvant therapy resulted in a higher incidence of increased aspartate aminotransferase (AST), dry skin, hepatitis, hyperthyroidism, hypothyroidism, infusion related reaction, pain, and pyrexia than ICI-free neoadjuvant therapy (**Table 2**). A total of 17 types of grade ≥3 AEs were available for meta-analysis. Grade ≥3 AEs including adrenal insufficiency, increased AST, hepatitis, and stomatitis were significantly increased by ICI-containing neoadjuvant therapy (**Table 2**).

**Table 2:** Meta-analysis for all grade and grade ≥3 adverse events.^a^

Adverse events	All grade			Grade > 3		
	No. of studies	OR (95% CI)	*P*-value	No. of studies	OR (95% CI)	*P*-value
Abdominal pain	3	1.50 (0.55–4.05)	0.43	NA		
Adrenal insufficiency	3	6.77 (0.42–108.65)	0.18	3	18.02 (2.36–137.48)	**0.005**
ALT increased	4	1.31 (0.89–1.91)	0.17	3	1.51 (0.80–2.87)	0.21
Alopecia	4	1.04 (0.85–1.26)	0.72	NA		
Anaemia	4	1.14 (0.80–1.61)	0.47	3	1.25 (0.94–1.68)	0.13
Anorexia	2	1.13 (0.67–1.91)	0.65	NA		
Arthralgia	3	1.03 (0.58–1.84)	0.92	NA		
AST increased	4	1.29 (1.01–1.66)	**0.04**	3	4.03 (1.40–11.63)	**0.01**
Asthenia	3	1.00 (0.78–1.27)	0.97	NA		
Back pain	3	0.89 (0.59–1.34)	0.59	NA		
Bone pain	2	0.84 (0.46–1.56)	0.59	NA		
Colitis	3	2.01 (0.69–5.81)	0.20	3	3.16 (0.72–13.97)	0.13
Constipation	4	1.06 (0.86–1.31)	0.58	NA		
Cough	3	1.25 (0.62–2.50)	0.53	NA		
Decreased appetite	3	1.17 (0.82–1.66)	0.39	NA		
Depression	2	1.37 (0.81–2.32)	0.24	NA		
Dermatitis	2	1.02 (0.48–2.19)	0.96	NA		
Diarrhoea	4	0.97 (0.64–1.48)	0.90	3	2.20 (0.92–5.28)	0.08
Dry eye	2	1.46 (0.77–2.78)	0.24	NA		
Dry skin	3	1.59 (1.04–2.43)	**0.03**	NA		
Dysgeusia	3	1.14 (0.69–1.88)	0.60	NA		
Dyspepsia	2	0.90 (0.54–1.51)	0.69	NA		
Dyspnea	3	1.43 (0.97–2.11)	0.07	NA		
Epistaxis	3	1.34 (0.92–1.94)	0.13	NA		
Fatigue	4	1.13 (0.92–1.38)	0.24	4	1.66 (0.56–4.96)	0.36
Febrile neutropenia	4	1.16 (0.90–1.50)	0.26	4	1.17 (0.88–1.55)	0.27
Headache	3	1.28 (0.92–1.78)	0.14	NA		
Hepatitis	4	3.20 (1.06–9.68)	**0.04**	4	7.37 (1.28–42.27)	**0.03**
Hot flush	3	1.19 (0.81–1.74)	0.37	NA		
Hyperglycemia	2	0.94 (0.34–2.61)	0.90	NA		
Hypertension	2	0.60 (0.30–1.22)	0.16	NA		
Hyperthyroidism	4	6.43 (2.75–15.03)	**<0.001**	NA		
Hypophysitis	2	7.04 (0.84–58.70)	0.07	NA		
Hypotension	2	4.36 (0.05–369.20)	0.52	NA		
Hypothyroidism	4	4.91 (2.94–8.19)	**<0.001**	NA		
Infection	2	0.73 (0.27–1.99)	0.54	NA		
Infusion related reaction	4	1.71 (1.26–2.33)	**<0.001**	3	2.24 (0.82–6.15)	0.12
Insomnia	2	1.36 (0.92–2.01)	0.13	NA		
Lacrimation increased	3	1.25 (0.70–2.22)	0.45	NA		
Leucopenia	3	0.91 (0.41–2.00)	0.81	NA		
Malaise	2	1.45 (0.32–6.44)	0.63	NA		
Myalgia	3	1.14 (0.66–1.99)	0.64	NA		
Nail discoloration	2	1.15 (0.56–2.34)	0.70	NA		
Nail disorder	2	0.79 (0.42–1.51)	0.48	NA		
Nausea	4	1.00 (0.82–1.22)	1.00	4	1.00 (0.13–7.70)	1.00
Neutropenia	4	1.10 (0.73–1.65)	0.66	4	1.04 (0.84–1.29)	0.73
Neutrophil count decreased	3	0.89 (0.66–1.21)	0.46	NA		
Edema	2	1.04 (0.35–3.07)	0.94	NA		
Edema peripheral	2	1.26 (0.71–2.24)	0.43	NA		
Oropharyngeal pain	2	1.11 (0.64–1.92)	0.71	NA		
Pain	2	1.74 (1.03–2.95)	**0.04**	NA		
Pain in extremity	2	1.00 (0.60–1.69)	0.99	NA		
Paresthesia	2	0.56 (0.23–1.35)	0.19	NA		
Paronychia	2	0.39 (0.17–0.90)	0.03	NA		
Peripheral Neuropathy	3	1.16 (0.74–1.82)	0.53	NA		
Peripheral sensory neuropathy	4	1.02 (0.82–1.28)	0.83	4	1.05 (0.57–1.93)	0.87
Pneumonitis	4	1.42 (0.63–3.20)	0.40	4	1.56 (0.31–7.77)	0.59
Pruritus	2	1.93 (0.65–5.69)	0.23	2	0.37 (0.06–2.29)	0.29
Pyrexia	3	1.79 (1.34–2.40)	**<0.001**	NA		
Rash	3	1.37 (0.95–1.96)	0.09	NA		
Stomatitis	4	1.23 (0.97–1.56)	0.09	4	5.78 (1.01–33.05)	**0.05**
Upper respiratory tract infection	2	1.08 (0.63–1.85)	0.77	NA		
Vertigo	2	0.90 (0.20–4.14)	0.90	NA		
Vomiting	4	1.21 (0.77–1.92)	0.41	4	1.66 (0.74–3.70)	0.22

ALT, Alanine aminotransferase; AST, Aspartate aminotransferase; OR, odd ratio; CI, confidence interval; NA, data were not available due to limited number of studies or events.

a All meta-analyses were conducted by random-effects model Bold values represent statistically significant (p < 0.05).

## Discussion

Several immunotherapeutic agents, including atezolizumab, avelumab, durvalumab, nivolumab, and pembrolizumab, are currently being investigated for the treatment of early and metastatic BC (17–19). This study focused on the effect of ICIs on the pCR rate in patients with early TNBC. Based on the four latest RCTs (11, 12, 15, 16), the addition of ICIs to neoadjuvant chemotherapy significantly increase the pCR rate compared with that in the control group in TNBC patients. Although the anti-PD-1 inhibitor (pembrolizumab) group achieved a significantly higher pCR rate against TNBC than control group in the both original RCTs (KEYNOTE-522 and I-SPY2), the pooled ORs of our meta-analysis were not statistically significant. However, the pCR rate of the anti-PD-1 inhibitor group tended to increase. We speculated that this inconsistency may have resulted from the clinical heterogeneity of the two RCTs. For instance, carboplatin was added to the regimen and pembrolizumab was administered for up to eight cycles in the KEYNOTE-522 trial (11), whereas the neoadjuvant regimen contained no carboplatin and only four cycles of pembrolizumab were administered in the I-SPY2 trial (12). In addition, the limited number of patients with TNBC may have diminished the statistical results in the I-SPY2 trial (12).

In subgroup analysis, ICI-containing neoadjuvant therapy significantly increased the pCR rate in both PD-L1-positive and -negative subgroups. Inconsistently, the IMpassion130 study reported that atezolizumab showed PFS and OS benefit for patients with advanced TNBC only in the PD-L1-positive cohort (7). The inconsistency may be due to the differences between early and metastatic TNBC, ICIs used, different PD-L1 detection methods, other potential targets of ICIs, or patient selection. In addition, it should be noted that the proportion of PD-L1-positive and -negative TNBC were different in the three RCTs. However, the results of this study was similar with a previous meta-analysis that patients with both PD-L1-positive and -negative advanced or metastatic cancers receiving ICIs were associated with a better OS than conventional agents (20). On the other hand, we also found that TNBC patients with PD-L1-positive tumors had a higher pCR rate than those with PD-L1-negative tumors not only in the ICI-containing group, but also in the ICI-free group. It indicated that, in addition to an acknowledged prognostic factor in BC (21), PD-L1 might be a potential biomarker for predicting the response to neoadjuvant chemotherapy.

In regards to survival outcomes, only EFS was reported by three of the four RCTs. The EFS involving disease progression, local or distant recurrence, development of a second primary tumor, or death were better in the ICI-containing group than the ICI-free group among patients with TNBC. In subgroup analysis, we found that the addition of pembrolizumab to neoadjuvant chemotherapy was significantly associated with better EFS than control group. However, in the KEYNOTE-522 trial (11), there were eight (1.0%) and three (0.9%) deaths during the follow-up period in the pembrolizumab-chemotherapy and placebo-chemotherapy group, respectively, and the difference was not significant. A recent single-arm, phase II trial regarding pembrolizumab for the treatment of metastatic TNBC and hormone receptor-positive/HER2-negative endocrine-refractory BC demonstrated that pembrolizumab dose not significantly improve the median PFS compared with historic controls (8). Nevertheless, we propose that the follow-up period should be prolonged to observe the long-term effect of ICIs on survival outcomes in TNBC patients. Taken together, there were limited results regarding the effects of ICIs on survival outcomes in TNBC patients. Several RCTs (e.g., NCT03051659, NCT03125902, NCT02819518, and NCT03841747) are ongoing to evaluate the efficacy of ICIs for early and advanced BC with different subtypes. An updated meta-analysis including the upcoming results and extended follow-up periods will be needed.

Endocrine dysfunctions, such as adrenal insufficiency, hypothyroidism, hyperthyroidism, hypophysitis, and insulin-deficient diabetes, are the most common immune-related AEs reported in clinical trials involving ICIs (22–24). Consistently, in the present meta-analysis, there were significantly higher incidences of all-grade hyperthyroidism and hypothyroidism and grade ≥3 adrenal insufficiency in the ICI-containing group than the ICI-free group. However, inconsistent with the findings of a previous meta-analysis focusing on anti-PD-1 drugs (24), this meta-analysis found that the addition of ICIs did not significantly increase the incidence of pneumonitis or colitis. The most common AEs of toxic effects (25–27) typically observed with chemotherapeutic agents were similar between ICI-containing and ICI-free groups, which including ALT increased, alopecia, anemia, decreased neutrophil count, febrile neutropenia, nausea, neutropenia, and vomiting. However, ICI-containing neoadjuvant therapy significantly associated with AST increased. In addition, ICI-containing neoadjuvant therapy was associated with greater risks of dry skin, hepatitis, infusion reaction, pyrexia, and stomatitis, which were also occurred in other malignancies (28–32). Although the pathogenesis of these ICI-related AEs remains unclear, the toxicity effects are manageable.

There were several limitations in this study that should be addressed. First, only four RCTs were included in this meta-analysis and the number of included patients was relatively small. Therefore, future meta-analyses including RCTs with many more participants are warranted to strengthen the results of this study. Second, there were several potential heterogeneities between the four RCTs, including the study design, treatment regimens, and PD-L1 detection methods, and definition of PD-L1 positivity, which may have negatively affected the pooled results. Third, considering the good prognosis of BC (33), the follow-up period of the four RCTs was relatively too short to observe the long-term survival benefits of ICIs, especially for OS. Finally, because the 95% CIs of the HR and *P*-value of EFS were not directly reported in the I-SPY2 trial (12), the related statistical data were indirectly calculated using the validated Parmar methods (14). It might diminish the accuracy of the pooled results. However, despite these limitations, this study, for the first time, summarized the efficacy and safety of adding ICIs to the neoadjuvant therapy for the treatment of early TNBC.

## Conclusions

The addition of ICIs to neoadjuvant chemotherapy significantly increased the pCR rate in TNBC patients, regardless of PD-L1 status. ICI-containing neoadjuvant therapy was significantly associated with better EFS than ICI-free neoadjuvant therapy in TNBC patients. Although ICIs increased the risks of several kinds of AEs, the toxicity effects were manageable. Future phase III RCTs with larger sample sizes and long-term follow-up periods are required to strengthen the present findings.

## Data Availability Statement

The original contributions presented in the study are included in the article/**Supplementary Material**. Further inquiries can be directed to the corresponding authors.

## Ethics Statement

Ethical review and approval was not required for the study on human participants in accordance with the local legislation and institutional requirements. Written informed consent for participation was not required for this study in accordance with the national legislation and the institutional requirements.

## Author Contributions

GR, HYL, and XY conceived and designed the study. YL, LX, and DY performed the literature search, data extraction, quality assessment of the included studies. YL, LX, and LX performed the statistical analysis. YL and LX wrote the paper. FL, HL, LG, and MW reviewed and edited the manuscript. All authors contributed to the article and approved the submitted version.

## Funding

This work was supported by the National Natural Science Foundation of China (No. 82103089).

## Conflict of Interest

The authors declare that the research was conducted in the absence of any commercial or financial relationships that could be construed as a potential conflict of interest.

## Publisher’s Note

All claims expressed in this article are solely those of the authors and do not necessarily represent those of their affiliated organizations, or those of the publisher, the editors and the reviewers. Any product that may be evaluated in this article, or claim that may be made by its manufacturer, is not guaranteed or endorsed by the publisher.

## Supplementary Material

Supplementary Figure S1 Risk of bias assessment on the included four RCTs. **(A)** Risk of bias summary. **(B)** Risk of bias graph.

Supplementary Figure S2 Forest plots of subgroup meta-analyses of pCR based on anti-PD-1 and anti-PD-L1 inhibitors. **(A)** Patients receiving anti-PD-1-containing neoadjuvant therapy compared with the control group. **(B)** Patients receiving anti-PD-L1-containing neoadjuvant therapy compared with the control group.

Supplementary Figure S3 Forest plots of subgroup meta-analyses for pCR based on treatment. **(A)** PD-L1-positive group compared with PD-L1-negative group in TNBC patients receiving ICI-containing neoadjuvant therapy. **(B)** PD-L1-positive group compared with PD-L1-negative group in TNBC patients receiving ICI-free neoadjuvant therapy.
