# Supplementary figures and images for: Efficacy and Safety of Adding Immune Checkpoint Inhibitors to Neoadjuvant Chemotherapy Against Triple-Negative Breast Cancer: A Meta-Analysis of Randomized Controlled Trials
Source: Front Oncol. 2021 Nov 29;11:657634. doi: 10.3389/fonc.2021.657634 (PMC8667776; doi:10.3389/fonc.2021.657634)

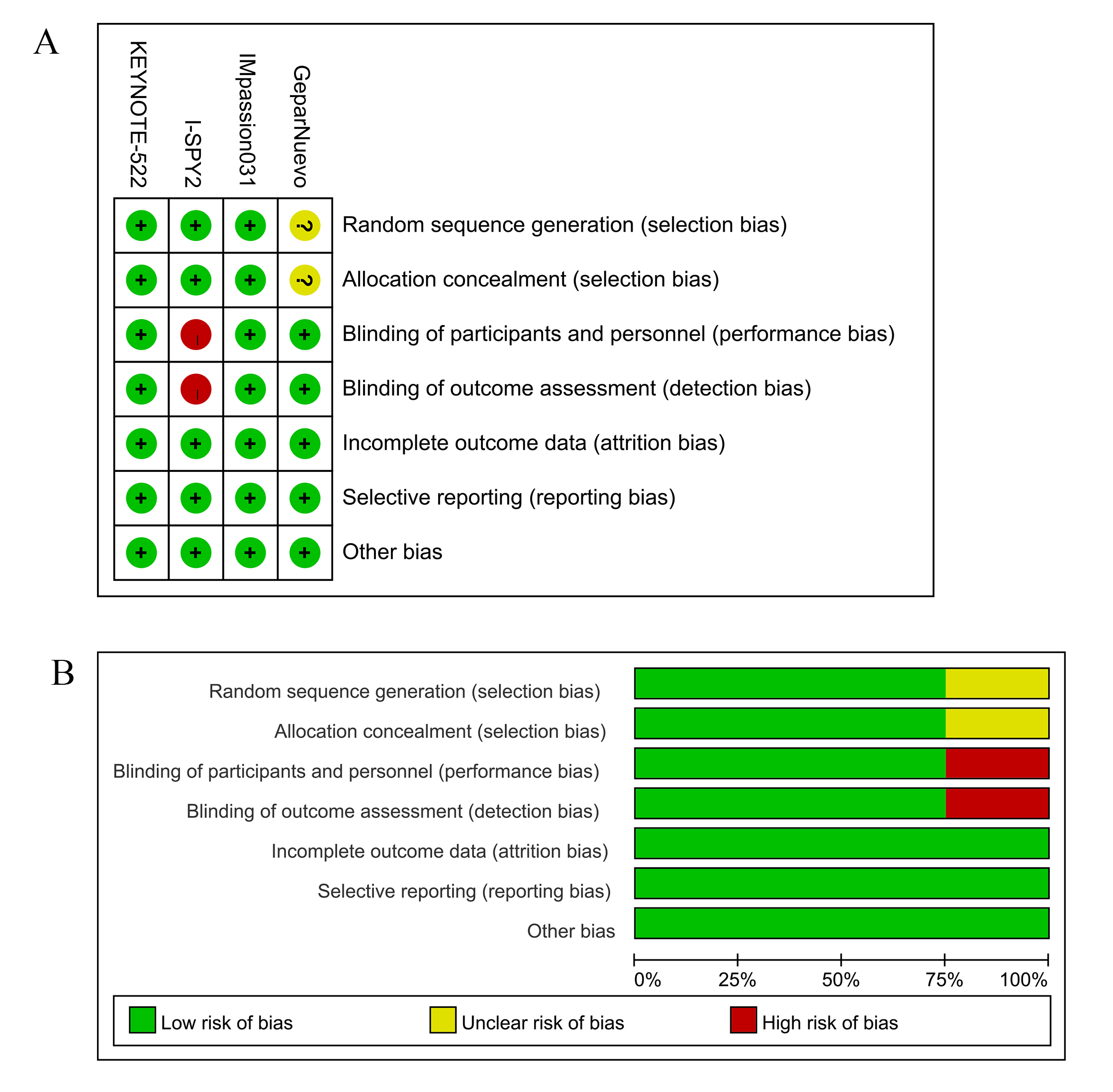

Supplement: Supplementary Figure S1 — Risk of bias assessment on the included four RCTs. (A) Risk of bias summary. (B) Risk of bias graph. [file Image_1.tif]

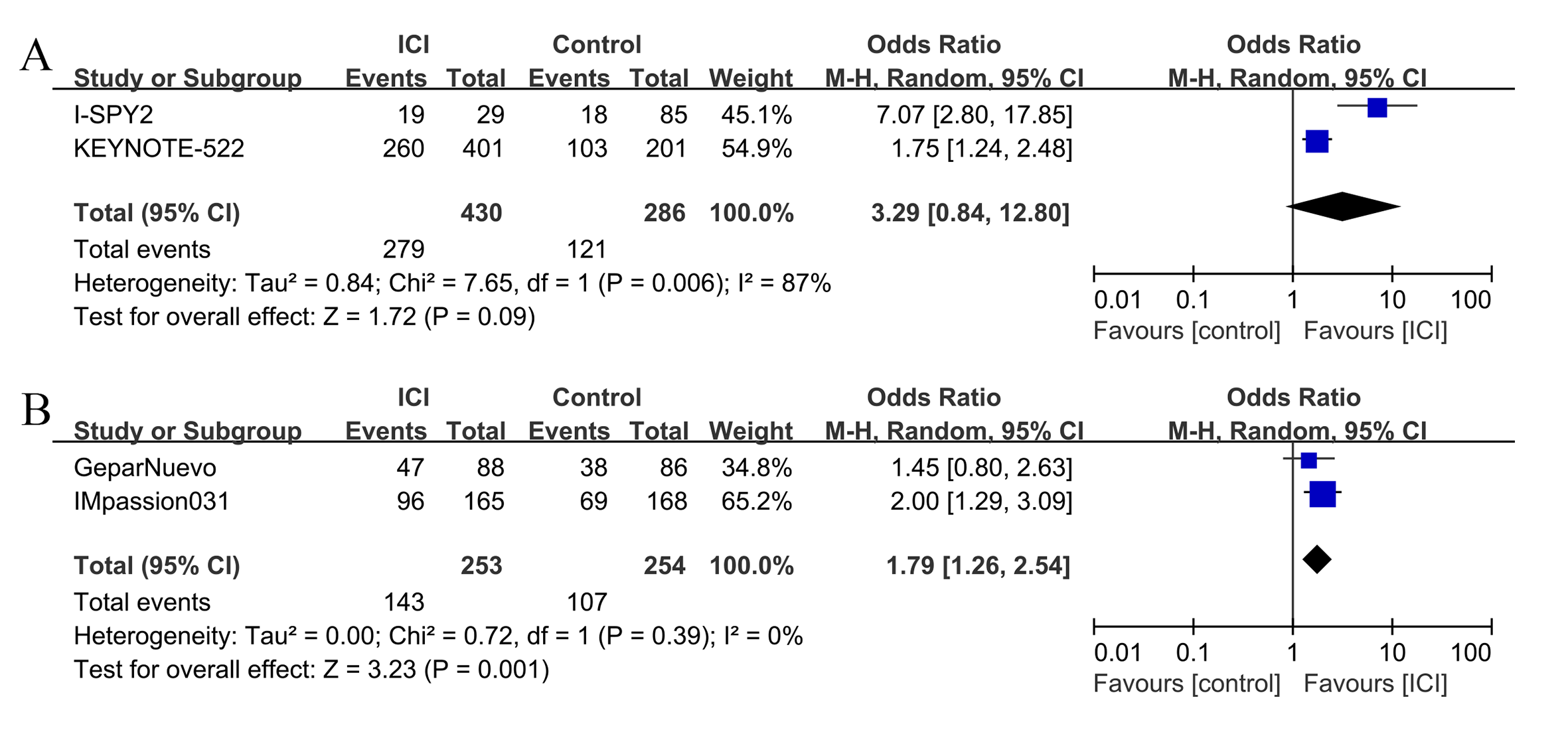

Supplement: Supplementary Figure S2 — Forest plots of subgroup meta-analyses of pCR based on anti-PD-1 and anti-PD-L1 inhibitors. (A) Patients receiving anti-PD-1-containing neoadjuvant therapy compared with the control group. (B) Patients receiving anti-PD-L1-containing neoadjuvant therapy compared with the control group. [file Image_2.tif]

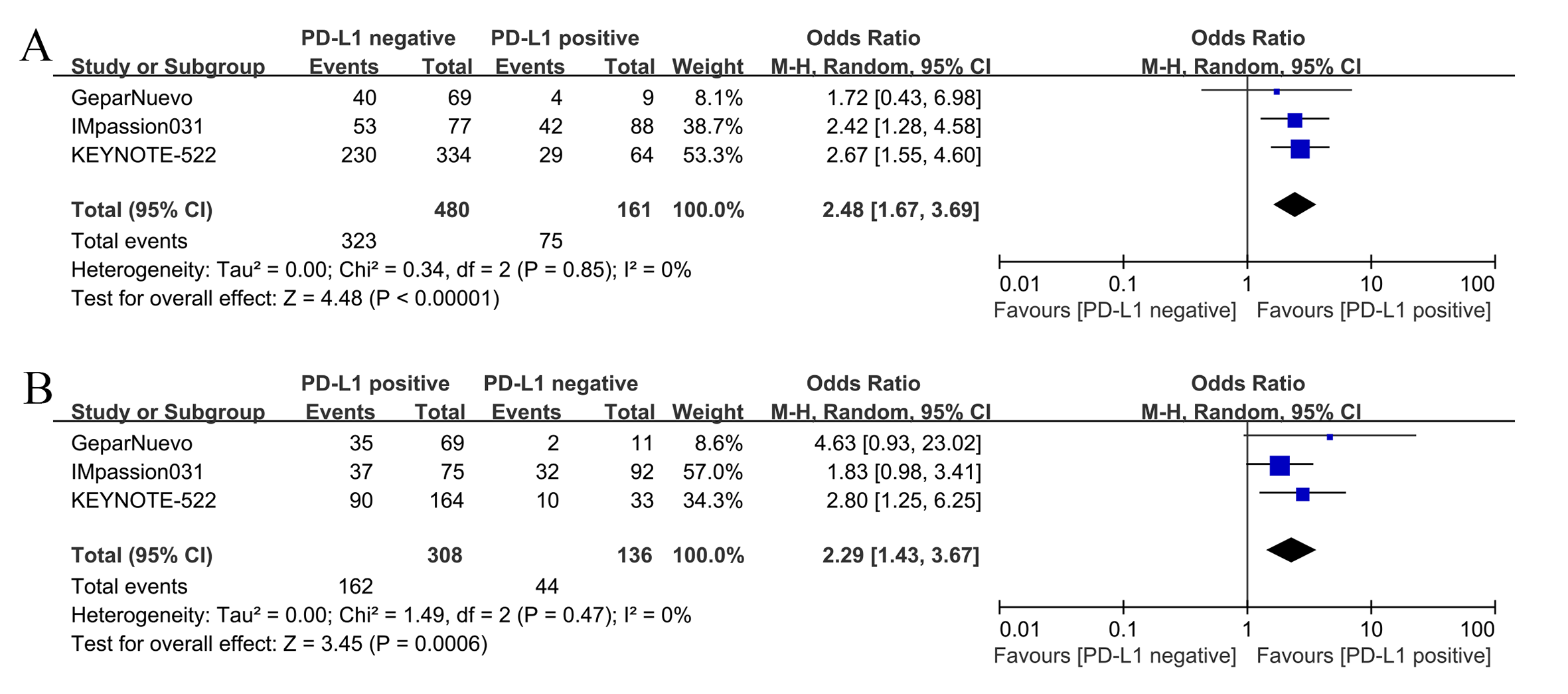

Supplement: Supplementary Figure S3 — Forest plots of subgroup meta-analyses for pCR based on treatment. (A) PD-L1-positive group compared with PD-L1-negative group in TNBC patients receiving ICI-containing neoadjuvant therapy. (B) PD-L1-positive group compared with PD-L1-negative group in TNBC patients receiving ICI-free neoadjuvant therapy. [file Image_3.tif]
